# Liver Bleeding Due to HELLP Syndrome Treated With Embolization and Liver Transplantation: A Case Report and Review of the Literature

**DOI:** 10.3389/fsurg.2021.774702

**Published:** 2021-11-22

**Authors:** Valentina Messina, Daniele Dondossola, Maria Chiara Paleari, Gianluca Fornoni, Daniela Tubiolo, Patrizia Vergani, Roberto Rona, Giorgio Rossi

**Affiliations:** ^1^Department of General and Liver Transplant Surgery Unit, Fondazione IRCCS Ca' Granda, Ospedale Maggiore Policlinico, Milan, Italy; ^2^Department of Pathophysiology and Transplantation, Università Degli Studi di Milano, Milan, Italy; ^3^Department of Anesthesia and Critical Care, Fondazione IRCCS Ca' Granda, Ospedale Maggiore Policlinico, Milan, Italy; ^4^Department of Maternal Fetal Medicine, Fondazione MBBM, San Gerardo Hospital, Monza, Italy; ^5^Department of Emergency and Intensive Care, University Hospital San Gerardo, Monza, Italy

**Keywords:** HELLP syndrome, liver transplantation, liver embolization, damage control surgery, rare disease

## Abstract

**Background:** Liver bleeding secondary to haemolysis, elevated liver enzymes, and low platelet count (HELLP) syndrome is uncommon, but a life-threatening peripartum condition that needs a prompt multidisciplinary approach.

**Case Presentation:** In this study, we presented a case of 28-year-old pregnant woman, who was presented to the obstetrics department with signs of preeclampsia and foetal growth restriction. An emergency caesarean section was performed, and the patient developed a HELLP syndrome complicated by spontaneous liver rupture. After radiological and surgical procedures, liver failure became evident and liver transplantation was successfully performed. The patient and her daughter are now alive.

**Conclusions:** Despite the rarity of this disease, liver complications due to HELLP syndrome must be properly diagnosed and treated given the gravity of the possible evolution in young women. After diagnosis, the patients must be treated in specialised centres with gynaecological, liver surgery, and transplant skills.

## Background

Haemolysis, elevated liver enzymes, and low platelet count (HELLP) syndrome is a severe uncommon complication of pregnancy (1). The risk of developing HELLP Syndrome is 0.5–1% but it occurs in 10–20% of pregnancies complicated by preeclampsia. It usually develops after week 27 and up to one-third of the cases can be diagnosed within 48 h post-partum. Multiparous women older than 30 years have an increased risk of developing HELLP during pregnancy (2). The mortality rate among women with HELLP syndrome is 0–24% and perinatal death rises up to 37% (3).

While the most frequent complication of HELLP Syndrome is acute kidney injury, surgical complications are weighted by the high rate of maternal and foetal death. Indeed, subcapsular liver haematoma could occur in patients affected by HELLP (3). This rare but potentially life-threatening condition is reported in 1:40,000 to 1:25,000 pregnancies (4). Spontaneous haematoma or liver rupture and liver ischaemia could complicate 0.9–2% of women with HELLP syndrome.

The exact aetiopathogenesis of the syndrome is not well known. It seems that a direct injury on the haepatocyte caused by placental factors, thrombotic microangiopathy, and the instauration of disseminated intravascular coagulation (DIC) may cause obstruction of haepatic sinusoids and haepatic infarction followed by subcapsular hematoma (5).

The management of this condition includes conservative treatment with supportive therapy, radiological intervention (artery embolization), or surgical procedures, such as packing, haepatectomy of the necrotic segments, haepatic artery ligation, laceration suturing. When haepatic failure with massive necrosis occurs, liver transplantation is the only treatment of choice for preserving life (6). For this reason, Gynaecologists, Emergency, and Liver surgeons should be aware of and properly manage this condition.

The aim of this case report is to describe successful multistep management of the haepatic rupture in HELLP syndrome.

## Case Presentation

**Figure 1:**
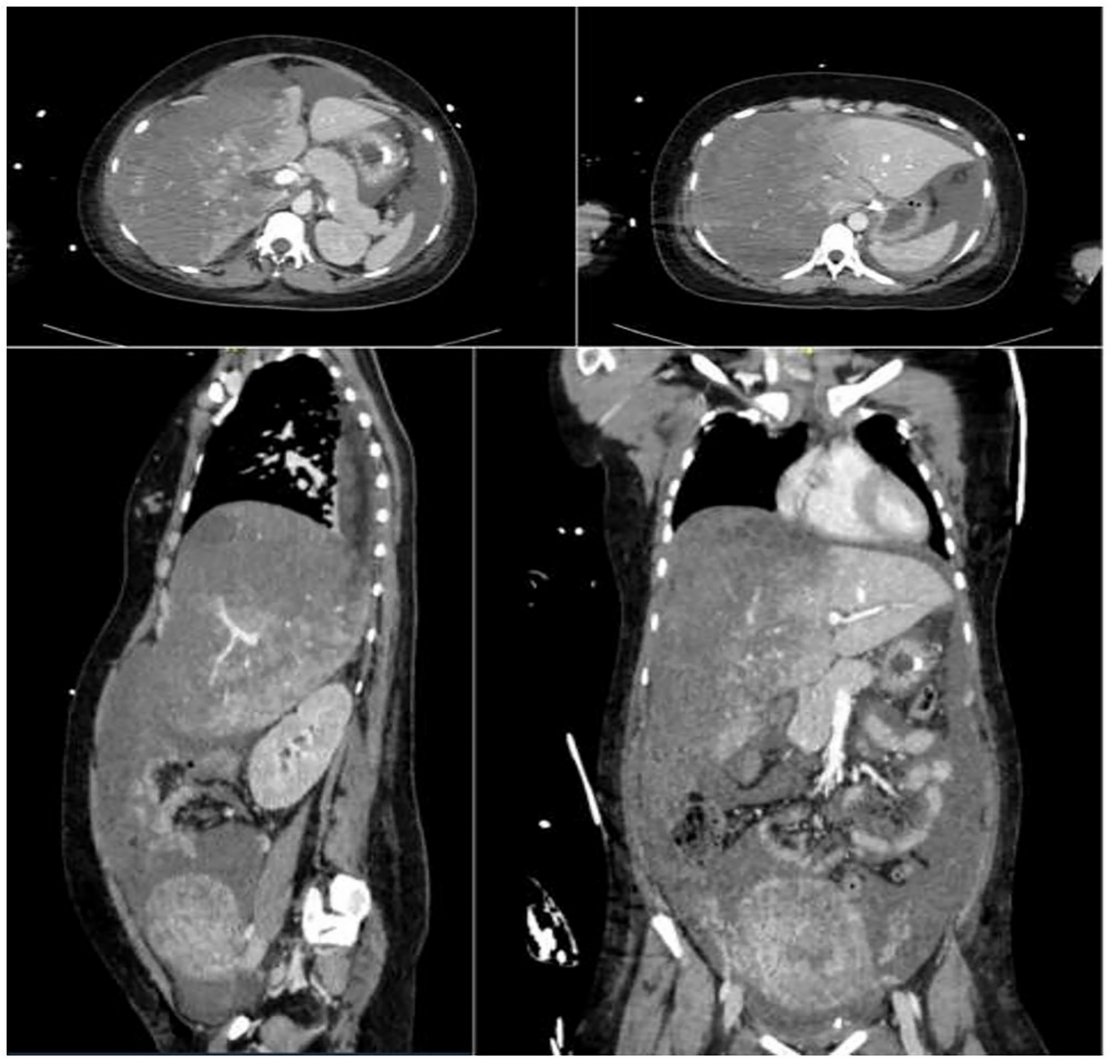
Computed tomography images of the large right liver haematoma with haemoperitoneum.

A 28-year-old nulliparous woman at 31+ 4 weeks gestation with no past medical history was admitted to the obstetrics and gynaecology (O/G) department of another hospital due to asymptomatic proteinuria, hypertension, and foetal growth restriction during a routine antenatal visit. Soon after hospitalisation, anti-hypertensive medication and steroid therapy for foetal lung maturation were started. A sudden mild epigastric pain was diagnosed after 36 h, together with worsening hypertension. Severe preeclampsia was diagnosed, and an ultrasound examination (US) suggested a placental abruption. For these reasons, she underwent a successful emergent caesarean section, and a healthy 1,500 g baby was born. The patient was transferred to the O/G department for post-partum care. The laboratory tests showed a progressive increase in liver enzymes with a concomitant reduction of platelet count and a HELLP Syndrome diagnosis was suggested (Table 1). The concomitant US did not reveal any pathological findings. A few hours later the patient suffered a haemorrhagic shock (blood hypotension, tachycardia, lactic acidosis, and anaemia). Then, the woman was admitted to the intensive care unit (ICU) and a massive transfusion protocol was successfully started (3 RBC units, 1,500 ml FFP, and 2 g fibrinogen). At the same time, haepatonecrosis markers peaked. An abdominal CT scan revealed a haemoperitoneum and a large subcapsular haematoma of the right lobe of the liver (Figure 1). Due to the absence of a liver surgery unit in the hospital and to the poor clinical conditions of the patient, an urgent radiological artery embolization of the right haepatic artery with Spongostan was performed.

**Table 1:** Blood test before delivery and at haemolysis, elevated liver enzymes, and low platelet count (HELLP) syndrome diagnosis.

	**Before delivery**	**HELLP diagnosis**
WBC	8.59^*^ 10^3^/mmc	13.3^*^ 10^3^/mmc
RBC	3.84^*^ 10^6^/mmc	2.27^*^ 10^6^/mmc
Hb	11.1 g/dL	8 g/dL
Ht	32.2%	23.9%
PLT	206^*^ 10^3^/mmc	63^*^ 10^3^/mmc
AST-GOT	16U/L	2622 U/L
ALT-GPT	9U/L	1606 U/L
Crea	0.6 mg/dL	1 mg/dL
LDH	198 U/L	3587 U/L

* *In the figure shows the right liver hematoma*.

**Figure 2:**
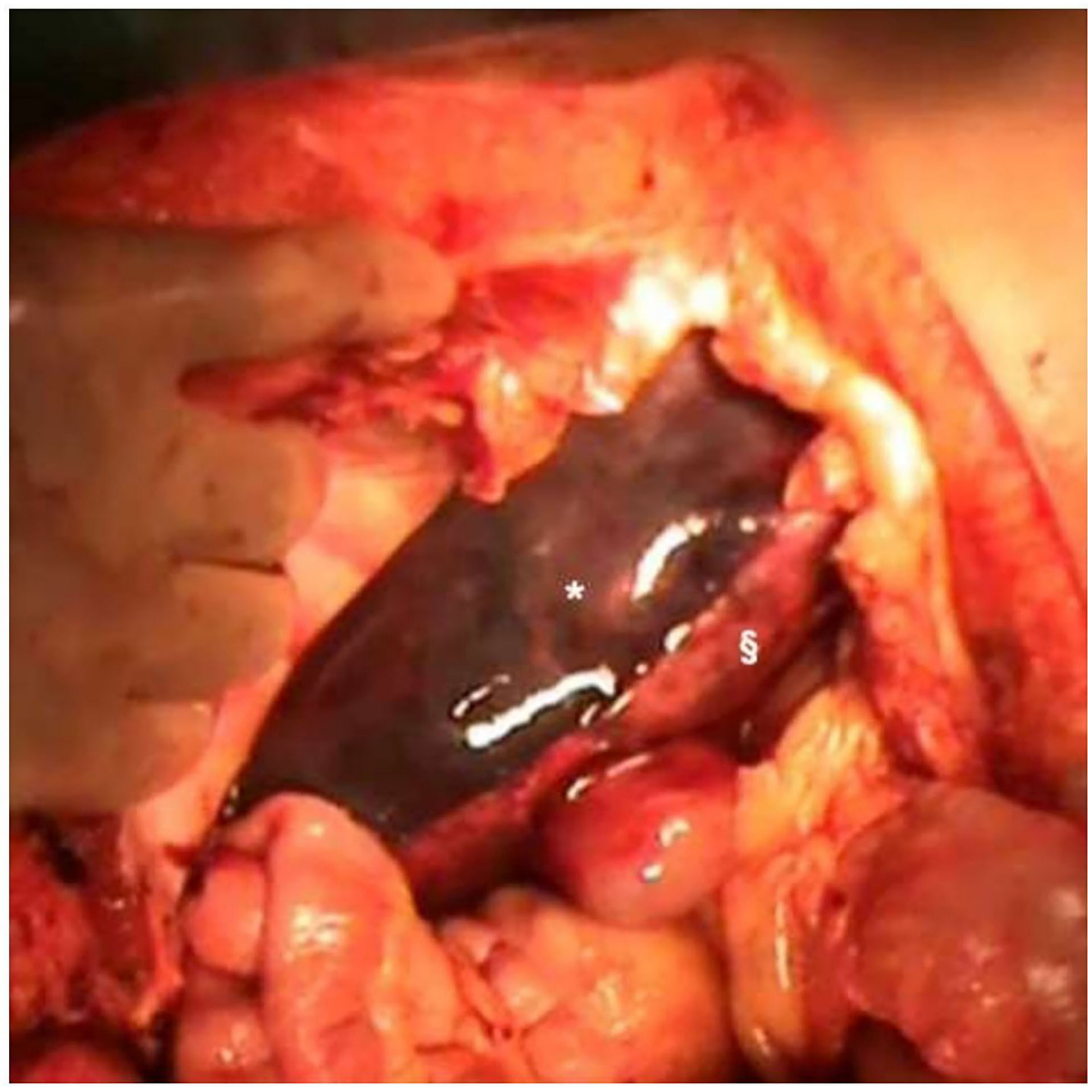
Right liver haematoma and liver ischaemia during the first laparotomy after haemoperitoneum evacuation. ^*^Right liver haematoma; ^§^Right liver ischaemia.

After 3 h, a further haemorrhagic shock was diagnosed with increasing haemoperitoneum. At that time, the General, and Liver Transplant Surgery Unit of Fondazione IRCCS Ca' Granda Ospedale Maggiore Policlinico Milan was contacted. After a multidisciplinary evaluation with ICU specialists, the patient was referred to our hospital. At ICU arrival, the woman showed signs of haemorrhagic shock with severe haepatic and renal impairment, and a massive transfusion protocol was started with shock resolution. However, the patient developed abdominal compartment syndrome (intra-abdominal pressure 35 mmHg) with the continuous need for blood component transfusion and an abdominal CT revealed a complete liver right lobe ischaemia with subcapsular haematoma and active bleeding. An urgent laparotomy was performed with evidence of haemoperitoneum, which has lacerated large subcapsular haematoma of the right lobe of the liver, without liver parenchymal rupture (Figure 2), but with complete necrosis of the right lobe. In order to control bleeding, under temporary Pringle manoeuvre, a total Glisson capsule removal of the right lobe was performed, and haemostasis was obtained with bipolar coagulation and fibrin glue. Due to massive liver necrosis with small future remnant liver volume (24%), post-haemorrhagic coagulopathy, and the conditions of the liver resection of the patient was not considered viable. For these reasons, liver transplantation (LT) was indicated, and right artery and portal ligation was performed, together with abdominal packing, to reduce liver bleeding. The abdomen was closed, the patient was listed for LT and transferred to the ICU while waiting for the LT.

In the following hours, the kidney of the patient and haepatic function worsened, with a third bleeding episode (four RBC units, four FFP units, and one pooled platelet). Then, a second-look laparotomy was performed at 24 h with the finding of a left lobe subcapsular haematoma that was treated as previously described. A few hours later, a compatible liver graft was allocated to our patient, and she was transplanted. The previous surgical procedures did not allow the caval preservation technique, so we opted for a termino-terminal caval anastomosis. Due to the aforementioned impairment of kidney function and the complete cava clamping, a continuous renal replacement therapy (CRRT) by venovenous techniques through a double-lumen dialysis catheter was settled.

After LT, the kidney function quickly recovered, with CRRT weaning in the postoperative days (POD) 4. Sepsis due to *Escherichia coli* urinary infection was diagnosed and successfully treated with Cefotaxime. Consistently, a herpes simplex infection was treated with Aciclovir. The woman was discharged on POD 33 with her daughter. The patient was in good condition and remained healthy after 6 months of follow-up.

Histological examination of the liver revealed massive haepatic necrosis with intraparenchymal and subcapsular haemorrhage.

## Discussion

We described a case of successful management of ruptured liver haematoma due to HELLP syndrome. The haepatic complication took place after an urgent caesarean section due to placental abruption. A radiological procedure followed by a surgical intervention was performed to stop bleeding, but, due to secondary liver ischaemia and failure, liver transplantation was needed. Complex multi-institutional management that involved gynaecologists, liver transplant and emergency surgeons, and ICU specialists led to the successful treatment of a rare but life-threatening post-partum complication.

Despite its rare presentation, liver complications secondary to HEELP syndrome must be recognised and properly treated by both gynaecologists and surgeons. The presentation (typically within 48 h post-partum) with non-specific symptoms (epigastric pain, worsening hypertension, and proteinuria) made the diagnosis difficult. In order to exclude haepatic haematoma or haepatic laceration, under clinical suspicions, an ultrasound must be considered as a first step even if weighted by low sensitivity and specificity (7). To improve its diagnostic potential and better evaluate the perfusion of liver parenchyma, CT can be performed. In our case, a US failed to diagnose liver haematoma and the diagnosis was achieved later with a second-level examination.

Diagnosis and management of complicated HELLP syndrome are not standardised and only evidence-based algorithms are published. After a diagnosis of liver complication with US or CT, unruptured haematomas can be handled with conservative treatment in an ICU service with blood component infusion and a close follow-up (4).

Conversely, in case of increasing/ruptured haematoma or liver fracture, resuscitation and coagulopathy correction should be achieved alongside bleeding control. Radiological interventions (artery embolization) could represent a valid treatment whenever the patient remains stable, and the necrosis contained (2). However, surgery is required in the majority of the haepatic rupture in HELLP syndrome. Grand'Maison et al. (8) published a case series of seven patients successfully treated with embolization. Among them, only three patients had haepatic embolization only, while the others needed further procedures such as right haepatectomy (because of an infected haematoma after embolization) or surgical packing. The management of our patient was particularly difficult due to the impossible definitive treatment of the patient in the first centre. In this case, the patient underwent an artery embolization to control the haemodynamic instability, given the necessity of moving her to our centre. However, artery occlusion possibly contributed to worsening the haepatic ischaemia and made the LT necessary due to liver failure.

Once the decompensation of a patient took place with continuous transfusion needs, urgent laparotomy is mandatory as in our case report. During laparotomy, several surgical procedures can be chosen to obtain haemostasis: temporary Pringle's manoeuvre packing of the liver, evacuating the haematoma, suturing the laceration, ligating one branch of the haepatic artery or portal vein (1, 14). We decided to ligate portal and haepatic right artery only after an established indication for liver transplantation. The haemodynamic instability, post-haemorrhagic coagulopathy, and extensive liver necrosis did not enable a safe liver resection. Indeed, in literature, haepatic resection is suggested only in the case of haemodynamic stability (12).

Urgent LT, if necessary preceded by total haepatectomy and temporary portocaval shunt, can be performed as the last therapeutic procedure in case of massive and persistent bleeding and extensive haepatic necrosis with an unfavourable remnant liver volume (7, 12, 14). An extensive review of the literature revealed 21 cases (22 including this report) of liver transplantation for HELLP syndrome (Table 2). The main indications for liver transplantation were a severe liver failure, liver necrosis, and uncontrollable bleeding. In 7 (32%) cases, haepatectomy and portocaval shunt were performed to reduce bleeding while waiting for a graft. The most common post-LT complication (up to 60%) is acute kidney injury suggesting the systemic involvement of HEELP syndrome. While in 2 cases the outcome is unknown, the rate of graft loss was 30% and all except two patients are alive. In our case liver transplantation turned out to be necessary because of the extended haepatic necrosis. Liver necrosis could be induced by HELLP syndrome itself and worsened by haepatic artery embolization. However, the radiological procedure enables the referral of the patient to a specialised centre and the successful treatment of a young mother.

**Table 2:** Summary of the published articles reporting cases of liver transplantation due to liver rupture or ischaemia due to HELLP syndrome.

**Authors**	**Year**	**Country**	**N^**°**^ patient in the serie/LT**	**Recipient age**	**Week of presentation**	**Delivery**	**Causes of LT**	**Other complications**	**N^**°**^ radiological procedure**	**Type**	**N^**°**^ surgical procedure pre-LT**	**Type**	**Anhepatic phase**	**Outcome**
Adam E. Mikolajczyk et al. (9)	2017	USA	1/1	30	32	C-section	Extensive necrosis, Haemorrhage, Subcapsular hematoma	Multi organ failure	0	No	O	No	No	Unknown
Varotti et al. (10)	2010	Italy	1/1	43	38		Necrosis, Hematoma	0	0	No	0	No	Portocaval shunt	Alive
Shames et al. (7)	1987–2003	USA	8/8	24–37^*^	35.5–36	Vaginal (1), C-section.	Necrosis, Hepatic rupture	Acute kidney injury (1), Respiratory failure (1), Sepsis (2)	0	No	3	Abdominal packing, Extended right epatectomy followed by Hepatectomy (1)	Portocaval shunt (1)	Dead (2) Alive (6) Retransplantation (2)
Wicke et al. (4)	2004	Germany	5/1	29	34	C-section	Hematoma, Haemorrhage	Acute kidney injury, Pleural effusion	0	No	2	Packing of the liver	No	Alive
P.-N. Descheemaeker (11)	2009	France	1/1	33	39	Vaginal	Rupture of the right lobe, Necrosis	Acute kidney injury	0	No	3	Packing of the liver	Portocaval shunt	Alive
Strate et al. (12)	2000	Germany	1/1	26	34	C-section	Hematoma, Rupture of right lobe, Necrosis	Acute kidney injury, Encephalopaty, Acute respiratory distress syndrome	0	No	1	Packing of the liver	No	Unknown
Zarrinpar et al. (13)	1964–2006	USA	8/8	23–37^*^		Vaginal (1), C-section (7)	Hematoma, Haemorrhage, Liver failure	Encephalopathy (7), Acute kidney injury (4), Disseminated intravascular coagulation (3), Respiratory failure (1)	2	Artery embolization	1	Hematoma evacuation	Portocaval shunt (5)	Biliary leaks (3) Reoperation (3) Retransplantation (2)
Current case (the current case report)	2019	Italy	1/1	28	31.5	C-section	Hematoma, Haemorrhage, Necrosis	Acute kidney injury, pleural effusion	1	Right hepatic artery embolization	2	Packing of the liver	No	Alive

* *Range*.

## Conclusion

Despite the rarity of this disease, liver complications due to HELLP syndrome must be properly diagnosed and treated given the gravity of the possible evolution in young women. After diagnosis, the patients must be treated in specialised centres with gynaecological, liver surgery, and transplant skills. These centres must be contacted as soon as possible to plan adequate treatments and referral procedures. Haepatic artery embolization could be adequate treatment, especially to stabilise the conditions of the patient, but a high risk of liver necrosis in HELLP syndrome must be considered. Consistently, liver transplantation should be considered if more conservative strategies have already failed.

## Data Availability Statement

The raw data supporting the conclusions of this article will be made available by the authors, without undue reservation.

## Author Contributions

VM has drafted and designed the work, obtained the data, searched, analyzed, and interpreted the literature. DD was a major contributor in writing the case and substantively revised it. MCP, GF, DT, PV, and RR has contributed to the conception. GR has importantly contributed to the conception. All authors read and approved the final manuscript.

## Conflict of Interest

The authors declare that the research was conducted in the absence of any commercial or financial relationships that could be construed as a potential conflictof interest.

## Publisher's Note

All claims expressed in this article are solely those of the authors and do not necessarily represent those of their affiliated organizations, or those of the publisher, the editors and the reviewers. Any product that may be evaluated in this article, or claim that may be made by its manufacturer, is not guaranteed or endorsed by the publisher.

## Abbreviations

HELLP: Elevated Liver enzymes and Low Platelet count
DIC: disseminated intravascular coagulation
O/G: obstetrics and gynaecology
US: ultrasound examination
ICU: intensive care unit
LT: liver transplantation
CRRT: continuous renal replacement therapy
POD: postoperative day.
